# *Data Analysis WorkbeNch* (*DAWN*)

**DOI:** 10.1107/S1600577515002283

**Published:** 2015-04-02

**Authors:** Mark Basham, Jacob Filik, Michael T. Wharmby, Peter C. Y. Chang, Baha El Kassaby, Matthew Gerring, Jun Aishima, Karl Levik, Bill C. A. Pulford, Irakli Sikharulidze, Duncan Sneddon, Matthew Webber, Sarnjeet S. Dhesi, Francesco Maccherozzi, Olof Svensson, Sandor Brockhauser, Gabor Náray, Alun W. Ashton

**Affiliations:** a Diamond Light Source Ltd, Diamond House, Harwell Science and Innovation Campus, Didcot, Oxfordshire OX11 0DE, UK; b European Synchrotron Radiation Facility (ESRF), 71 Rue des Martyrs, Grenoble 38000, France; c European Molecular Biology Laboratory (EMBL), 6 Rue Jules Horowitz, Grenoble 38042, France

**Keywords:** software, *DAWN*, visualisation, analysis

## Abstract

Synchrotron light source facilities worldwide generate terabytes of data in numerous incompatible data formats from a wide range of experiment types. The *Data Analysis WorkbeNch* (*DAWN*) was developed to address the challenge of providing a single visualization and analysis platform for data from any synchrotron experiment (including single-crystal and powder diffraction, tomography and spectroscopy), whilst also being sufficiently extensible for new specific use case analysis environments to be incorporated (*e.g.* ARPES, PEEM). In this work, the history and current state of *DAWN* are presented, with two case studies to demonstrate specific functionality. The first is an example of a data processing and reduction problem using the generic tools, whilst the second shows how these tools can be targeted to a specific scientific area.

## Abstract 2

*DAWN* is a generic data analysis software platform that has been developed for use at synchrotron beamlines for data visualization and analysis. Its generic design makes it suitable for use in a range of scientific and engineering applications.

## 1. Introduction   

Given the brilliance of third-generation synchrotron sources and the consequent diversity of possible experimental set-ups it is perhaps unsurprising that the scientific data produced come in a huge variety of formats and sizes (Willmott, 2011 ▶). The primary role of these facilities is to allow users to analyse samples on state-of-the-art experiments and leave with data that can easily be processed and prepared for publication. It is key that the data collection, visualization and analysis methods provided by these facilities are simple and consistent, allowing barrier-less access to the sometimes highly complex experiments of a broad multi-disciplinary user base.

The *Data Analysis WorkbeNch* (*DAWN*) is an open-source multiplatform software package free to download and intended to supply easy data visualization and processing to users of synchrotron facilities. Produced as part of a collaboration between Diamond Light Source (DLS), the European Synchrotron Radiation Facility (ESRF) and the European Molecular Biology Laboratory (EMBL, Grenoble), *DAWN* is now integral to the analysis pipelines of many beamlines operated by these facilities. The list of functionality available in the software package is still growing and currently includes data reduction for small-angle X-ray scattering (SAXS); optimization of tomographic reconstruction; alignment and reduction for photo-emission electron microscopy (PEEM); and design of macromolecular X-ray diffraction experiment workflows (Brockhauser *et al.*, 2012 ▶). At Diamond, *DAWN* is tightly integrated into the acquisition software (*Generic Data Acquisition; GDA*) (Enderby & Pulford, 2004 ▶; Gibbons, 2008 ▶), the aim being to give users a consistent data visualization experience across beamlines that they can take home and use after their visit or even download and learn prior to arrival.

Herein we describe the purpose and history of the *DAWN* project, its architecture and illustrate the application of the software through two specific examples, which highlight the advantages of *DAWN* for a wider variety of scientific and engineering applications, both at synchrotron beamlines and more generally.

## 2. *DAWN* history   

The *DAWN* project developed from the merger of two complementary data visualization and analysis projects, *Scientific Data Analysis* (*SDA*) and *Data Analysis WorkBench* (*DAWB*), both of which were built using the same core technologies of the Java programming language (Arnold *et al.*, 1995 ▶, 2005 ▶) and the Java-derived Eclipse Rich Client Platform (Eclipse RCP) (Eclipse Foundation, 2004 ▶; McAffer & Aniszczyk, 2010 ▶). *SDA* was produced at Diamond in 2010, using visualization components which became available as part of the process of *GDA* changing to the modular Eclipse RCP structure. *DAWB* was developed in the same year, on the back of a long history of Eclipse RCP development at the ESRF, but based on *GDA*’s dataset model allowing the user to view data, set up experimental workflows and access analysis routines written in Python. The source code of these two projects was merged in early 2012 forming the initial version of *DAWN*. The EMBL became involved as part of the BioStruct-X project to produce a tool to improve the visualization of macromolecular crystallography data.

## 3. *DAWN* design   

### 3.1. Perspectives: partitioning of functionality   

For a single piece of software to perform such a wide variety of operations it is fundamentally important for it to be well structured, with different functions clearly partitioned. User experience would suffer greatly if all visualization tools were accessible at all times, irrespective of the task the user is trying to perform.

To overcome this issue, *DAWN* uses the Eclipse RCP concept of ‘perspectives’, which only present users with options pertinent to the analysis they wish to do. A perspective is effectively a miniature program designed to do a specific set of tasks. *DAWN* itself behaves like an empty container, the functionality of which is determined by the individual perspectives running within it. Table 1 ▶ contains the names and descriptions of the main perspectives in *DAWN*1.7.^1^

**Figure 1:**
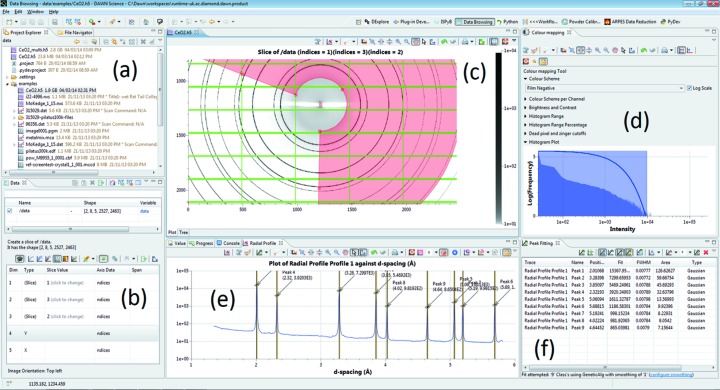
Screenshot of the *DAWN* Data Browsing perspective showing the radial profile of a powder diffraction image being peak fitted. The labelled components are: (*a*) the Project Explorer, for keeping track of files of interest; (*b*) the Data view, for selecting datasets or slices of datasets for display; (*c*) a plot of the selected data slice; (*d*) the colour mapping tool for adjusting the image contrast; (*e*) result of the radial integration tool (over the region specified by the red sector); and (*f*) the result of using the peak fitting tool to identify the peaks in the radial profile, and display their parameters.

By default, *DAWN* starts with the Data Browsing perspective (Fig. 1 ▶). This perspective allows a file containing one-dimensional traces, two-dimensional images or data with an arbitrary number of dimensions to be opened, datasets to be selected and, if desired, a slice (a subset of a dataset) selected for viewing. Basic analysis may also be performed using this perspective such as peak finding, fitting of expression to data or simple reduction along one dimension.

### 3.2. Data and file formats   

The synchrotron community is widespread with hundreds of separate beamlines around the world, many with different detectors and data formats. Although work has been done to make standardized formats available (Bicarregui, 2004 ▶) [*e.g.* NeXus (Klosowski *et al.*, 1997 ▶), CIF (Brown & McMahon, 2002 ▶; IUCR, 2006 ▶), *etc*.], there remains a lot of data in ASCII, image-based and other legacy formats. To support the great diversity of existing data formats, *DAWN* uses a modular plugin approach, allowing the addition of new data and metadata types without the need to rebuild the entire program. If a suitable plugin is available for a file, data are then loaded into one of *DAWN*’s generic dataset classes (see supporting information 2 for details of the architecture of the dataset).

Large multi-dimensional datasets can be a problem for many analysis packages. *DAWN* simplifies reading of such data through the concept of *lazy loading*. Lazy loading does not read the complete dataset into memory, but instead reads only a user-selected section or slice. For example, from a large imaging experiment (see Case Study I[Sec sec4.1]), the user might select one or a range of images to view and analyse from a single HDF5 file containing hundreds, if not thousands, of images (HDF Group, 2000 ▶; Folk & Pourmal, 2010 ▶). Without lazy loading it would be unfeasible to view such a dataset without very high-end computing resources. Lazy loading strikes a balance between storing data in memory (fast, but limited) and reading from disk (slower) depending on the specifications of the computer. *DAWN* is optimized for viewing data from the NeXus/HDF5 combination, taking full advantage of the rich, powerful and open platform defined by this standard and format.

### 3.3. The plotting system   

With data visualization being a key part of *DAWN*’s functionality, the plotting system is a vital component to its architecture and is used throughout the package, whenever data need to be displayed, giving a consistent experience for the user. Moreover, the plotting system is also used by the data acquisition software at Diamond, *GDA* (Gibbons, 2008 ▶), giving the user a consistent experience to the user throughout data collection and analysis. The plot supports most standard viewing modes (for example line graphs, scatter plots, images and surfaces) and allows basic customization, such as changing the colour of plot elements (lines, axes, titles *etc*.), manipulation of axes and changing or adding of axis titles. Plots may also be annotated with labels and specific regions may be selected (using a range of shape selection tools including lines, rectangles and sectors) either as an annotation or for specific analysis. Various colourmaps are available to tailor how images are displayed, and there are several different algorithms to map the data values onto the selected colourmap.

The plotting system is further extended by a variety of tools for processing the plotted data, depending on how the data is being displayed. For line graphs these include peak and function fitting, viewing the derivative of the data, as well as tools for specific science fields [such as X-ray absorption fluorescence spectroscopy (XAFS) and SAXS]. Images can be contrast/brightness adjusted, masked or profiled using the various different selection regions. The line traces generated by the image profile tools can then be further analysed using the line graph tool set (Fig. 1 ▶).

The plotting system has a well defined encapsulated Application Programming Interface (API; see supporting information) which does not expose the implementation of the plotting routines to the ‘outside world’ of mathematical algorithms or user interface. This means that the actual technology used to plot lines, images and surfaces is separated, allowing it to be swapped without changing core parts of the product. As with file loaders, this modular approach makes the plotting tool system easily extensible, allowing bespoke data visualization/analysis methods to be written and included in *DAWN*. It is the combination of the modular loading system, generic datasets, plotting system and plot tools that make *DAWN* a flexible and customizable piece of scientific software.

## 4. Case studies   

This section details two user experiments where the use of *DAWN* was key to the data analysis. The first was a radiography experiment studying the corrosion of metal as a function of time, where the data processing could be performed completely by using the simple tools in the Data Browsing perspective. The second example illustrates building a custom perspective to fulfil the needs of a very specific experiment (photo-emission electron microscopy), where re-use of the core *DAWN* framework reduced both the effort required to produce the necessary functionality and support costs compared with writing a standalone program from scratch.

### 4.1. Case Study I: calculating corrected corrosion rates with time-resolved radiography   

Time-resolved radiography experiments are common at synchrotrons, where the high flux of X-rays allows high-speed imaging (Rau *et al.*, 2011 ▶). This case study focuses on the processing of data collected to study the corrosion of metals under salt solutions (a video to accompany this case study is available^3^). The radiography presented here captures this corrosion as it occurs; quantitative analysis is used to determine the rates of corrosion.

While none of the data reduction or processing steps in this radiography experiment are particularly complex, the sheer volume of data means the analysis usually requires use of a scripting language such as MATLAB or Python, adding a significant barrier for a user who is unfamiliar with programming. The raw experimental data consist of 3600 images (30 gigabytes), whilst a further two datasets (or ‘stacks’) of 30 images each are needed to perform flatfield and darkfield corrections on the raw data, in order to improve the signal-to-noise ratio. All of the required datasets are collected as NeXus files, so will be read using lazy loading in *DAWN*, removing the usual memory issues that occur with such large sizes of files.

**Figure 2:**
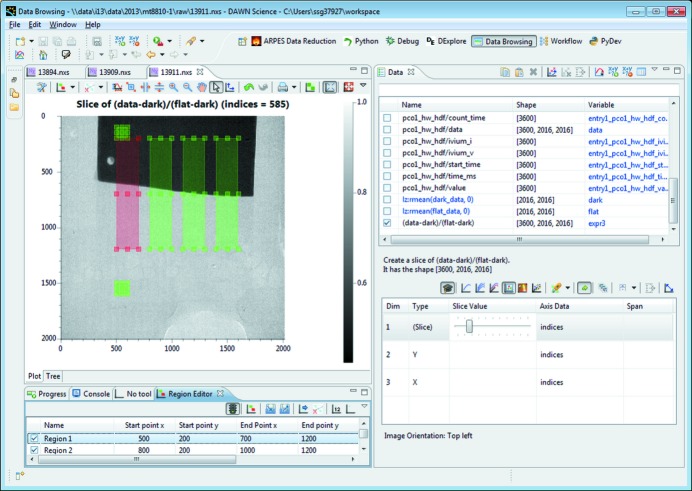
The Data Browsing perspective. In this case the data window has been used to run mathematical expressions on the data to produce the corrected images, as shown. The image and the view at the bottom left show the region editor tool for selecting regions of interest, which are used in the data reduction of this sample (shown in red and green).

The averaging of the flatfield and darkfield stacks, as well as the correction of the raw data, can be performed using the Data Browsing expression parser (Fig. 2 ▶, top right). This tool allows simple mathematical operations to be performed on arbitrary data, including all the steps required here; *e.g.* averaging, subtraction and division. Means are taken of the flatfield and darkfield stacks and these are then used to perform flatfield and darkfield corrections to the main stack of 3600 images (Seibert *et al.*, 1998 ▶). *DAWN* only corrects the currently displayed image, allowing the user to visually check that the operation is performed correctly, before applying it to the full set of data.

The region editor tool (shown in Fig. 2 ▶, left) is used to select four regions in which the rate of corrosion will be investigated and two control regions where no corrosion will occur. For all six regions, the *X* and *Y* profiles are determined and their means and standard deviations are calculated. This complete series of operations, starting with the corrections, is then applied to the entire stack using the data reduction tool and the result is output as a new NeXus file. As a final step, the expression parser is used to normalize the data in the newly created NeXus file, based on the control regions. Rates of corrosion are then determined from the normalized reduced data using the line-fitting tool.

### 4.2. Case Study II: image stack alignment and domain-specific processing for PEEM   

*DAWN* comes with a wide range of generic tools for processing and analysing data. However, many experiments require specialist analysis routines for which these tools are not intended. In cases where these routines would be applied numerous times, instead of requiring a new program to be developed, *DAWN* provides a simpler option. Thanks to its modular nature, the generic components in *DAWN* can be combined into a new perspective along with bespoke analysis code and user interface elements to form a new analysis tool.

**Figure 3:**
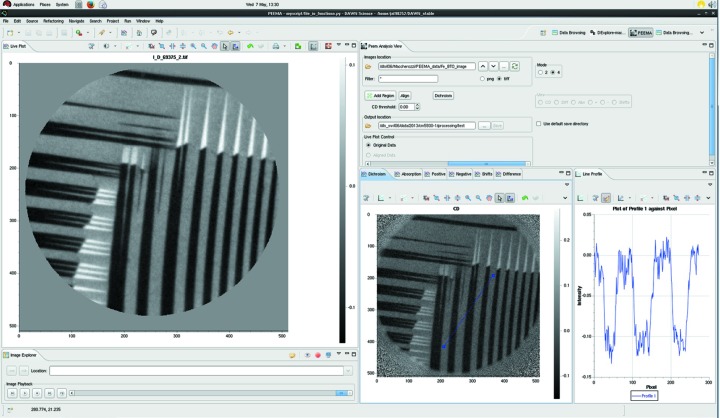
The PEEMA perspective. The custom PEEM analysis view is situated at the top right of the screenshot, with the other images, colour mapping tool and image explorer being generic views which have been reused.

This case study details the specific use case at beamline I06 (Diamond Light Source, UK), where PEEM is used to conduct X-ray magnetic circular dichroism (XMCD) experiments (a video to accompany this case study is available^4^). A custom perspective was developed for the beamline which aims to streamline the process of data reduction as much as possible, whilst requiring the minimum amount of new code and on-going support. In the experiment several exposures of a sample are taken whilst the polarization and energy of the illuminating X-ray beam are varied. By averaging and combining these images it is possible to retrieve the magnetic domain configuration of the sample surface. Often multiple images are taken at each condition to improve the signal-to-noise ratio, but, as the technique focuses on the nanoscale, these images are often subject to sample drift, so require aligning before processing can be done. *DAWN*’s PEEM Analysis (PEEMA, Fig. 3 ▶) perspective allows multiple images recorded with different X-ray polarizations and energies to be processed for data evaluation in a few mouse clicks, with processing complete in less than a second. As these experiments are routinely performed on a variety of samples, having user-friendly and efficient methods for analysing the data is paramount to the smooth and efficient running of the beamline.

*DAWN* facilitates development of such specialist environments through re-use of existing components and simple creation of new ones. The advantage of component re-use is that it dramatically reduces the development time of custom interfaces, whilst new features and bug fixes become available with limited additional development time. Core routines such as data access (loading and saving), data plotting and basic processing methods (for example, sum, mean) are already provided by *DAWN*. The standard plotting system is feature rich and thus histogram/colour mapping and region selection tools come as standard. To facilitate the required image alignment, bespoke routines were written and incorporated into the *DAWN* source code as a core component. The main algorithm which was required for this work was the alignment of images, which was implemented using standard Fourier methods (Foroosh *et al.*, 2002 ▶). This approach of extending *DAWN*’s basic functionality rather than adding custom code is taken with all generic processing tools, whenever possible. The ‘PEEM Analysis View’, which supplies the user with a pre-determined workflow for the analysis of the I06 beamline data, is the only custom graphical component in this perspective. It is there to provide a single location for the user to decide which files to load, what processing needs to be done, and where to save the result.

## 5. Conclusion   

*DAWN* is a versatile and extensible piece of software, which makes use of a number of existing technologies and libraries to produce a highly functional data analysis tool. *DAWN* is easy to install and runs on the majority of modern operating systems. A deliberate effort was made not to develop the software for a specific field or technique in order for it to remain a generic tool. As a result, interest from parties outside the synchrotron community is growing, examples of which include visualizing simulations of nuclear reactors and providing a data visualization tool for commercial X-ray detectors. It has also been shown that the framework can be extended easily to deal with bespoke analysis tasks, whilst the feature-rich generic tools can be rapidly customized to provide a clean user experience for repetitive analysis tasks.

## 6. Related literature   

The following references are mentioned in the Supporting Information: Jones *et al.* (2001 ▶); Walt *et al.* (2011 ▶); Hugunin *et al.* (undated ▶); Pedroni & Rappin (2002 ▶); Chang (2010 ▶).

## 7. Author contributions   

MB, JF, PCYC, BEK, MG, JA, KL, IS, DS, OS and GN collaboratively developed the *DAWN* software package, whilst MW maintained the compilation and testing environments. MB, JF and MTW jointly prepared the text for this article. SD and FM provided data from their beamline and conceptual input for the PEEMA case study. AWA, BCAP and SB directed the development effort and the writing of this article.

## Supplementary Material

Sections S1: DAWN User and Developer Resources; and S2: DAWN Technical Information.. DOI: 10.1107/S1600577515002283/fv5032sup1.pdf

## Figures and Tables

**Table 1:** List of perspectives available by default within *DAWN*1.7 and their function

Perspective name	Function/description
Data Browsing	The data browsing perspective is suitable for viewing line traces, images and multidimensional data. It also contains more advanced features including the ability to apply a tool to a stack of images, and mathematical processing of data with expressions.
DEXPLORE	An advanced alternative to data browsing. Provides many additional options for how data are shown; it also contains features for comparing data from multiple files as well as connecting the plots to a Python/Jython Interpreter.
Python/Jython Scripting	Provides a PyDev (Zadrozny, 2003 ▶) environment for opening, editing and displaying Python source files.
Tomography Reconstruction	Tools to access reconstruction routines for NeXus tomography data (requires additional software).
Workflows	Designing scientific algorithms with a graph-like structure, similar to LabView (Elliott *et al.*, 2007 ▶), implemented using Passerelle (Brockhauser *et al.*, 2012 ▶).
Trace	For working with line traces from multiple files.
dViewer	For viewing two-dimensional images from diffraction experiments. Includes features for highlighting spots and summing a range of images.
Powder Diffraction Calibration	A tool to calibrate two-dimensional powder diffraction images.
ISPyB	Communicates with facility ISPyB database of experiments (Delagenire *et al.*, 2011 ▶).
PEEMA	Analysis of PEEM data.
MX Live Analysis Overview	Permits monitoring of the current state of auto-processing on Diamond beamlines.

## Footnotes

1
*DAWN*1.7 is freely available for download from http://www.dawnsci.org and also from doi:10.5281/zenodo.14084.

2 Supporting information for this paper is available from the IUCr electronic archives (Reference: FV5032).

3 The video for Case Study I[Sec sec4.1] (Radiography) is available at doi:10.5281/zenodo.12473.

4 The video for Case Study II[Sec sec4.2] (PEEMA) is available at doi:10.5281/zenodo.12499.
