# Supplementary material for: Data Analysis WorkbeNch (DAWN)
Source: J Synchrotron Radiat. 2015 Apr 2;22(Pt 3):853–8. doi: 10.1107/S1600577515002283 (PMC4416692; doi:10.1107/S1600577515002283)
Supplement: Supplementary file 1 [file s-22-00853-sup1.pdf]

JOURNAL OF SYNCHROTRON RADIATION

Volume 22 (2015)

Supporting information for article:

Data Analysis WorkbeNch (*DAWN*)

Mark Basham, Jacob Filik, Michael T. Wharmby, Peter C. Y. Chang, Baha El Kassaby, Matthew Gerring, Jun Aishima, Karl Levik, Bill C. A. Pulford, Irakli Sikharulidze, Duncan Sneddon, Matthew Webber, Sarnjeet S. Dhesi, Francesco Maccherozzi, Olof Svensson, Sandor Brockhauser, Gabor Náray and Alun W. Ashton

S1. **DAWN User and Developer Resources**

DAWN is under active development with new requirements being submitted and new features being added all the time. As such the majority of documentation is available online, with additional developer documentation present within the code itself. For new users of the software, a range of training resources are available including:

- The DAWN website:

  http://www.dawnsci.org/

- Tutorials:

  http://confluence.diamond.ac.uk/display/DT/DAWN+Training+Home

- DAWN YouTube channel:

  https://www.youtube.com/user/DAWNScience

- Mailing list:

  https://www.jiscmail.ac.uk/DAWN

A new developer who would like to be involved in extending DAWN and contributing to the project would be directed to the more technical information. Experience of Java/Eclipse RCP or Python/Jython programming would be required. APIs and documentation are available to download:

- The DAWN GitHub repositories:

  https://github.com/DawnScience

- Developer documentation:

  http://www.dawnsci.org/documentation

- Developer mailing list:

  https://www.jiscmail.ac.uk/DAWN-DEV

DAWN is developed to the Java 7 standard (as of v. 1.7) and uses the eclipse 3 RCP framework.

S2. **DAWN Technical Information**

DAWN is written in Java,(Arnold et al., 1995, 2005) with the user interface based on the Eclipse Framework.(Eclipse Foundation, 2004; McAffer & Aniszczyk, 2010) This framework provides a very modular architecture, and rich set of core features which simplify and streamline the development process. DAWN scales to make use of all available processor cores on the machine, so it works on laptops as well as the high end workstations found on beamlines. Installation is done by simply unzipping the downloaded file, and then running the extracted executable, which requires no additional administrator privilege. DAWN is packaged with a local Java virtual machine which does not need to be installed separately.

Large multi-dimensional datasets can be a problem for many analysis packages. DAWN simplifies reading of such data through the concept of *lazy datasets*. Lazy datasets do not load the complete dataset into memory, but instead load only a user selected section or slice of the data. For example, from a large imaging experiment (see Case Study I), the user might select one or a range of images to view and analyse from a single HDF5 file containing hundreds, if not thousands, of images.(HDF Group, 2000; Folk & Pourmal, 2010) Furthermore, the lazy loading framework can be accessed by any custom loader plugin, simply by implementing the correct interface.

Once the file or slice has been accessed, data is read into the generic dataset container. The `IDataset` interface together with its implementation, `Dataset`, simplifies the writing of mathematical methods within the DAWN Java source code, as it allows mathematical operations to be performed without prior knowledge of the data type (e.g. integer, float or double). Thus datasets may be manipulated in a similar fashion to dynamically typed programming languages such as Python or MatLab. Indeed, the DAWN source code includes a broad range of common mathematical algorithms for data processing.

The behaviour of the `Dataset` class is similar to `ndarray` class of the NumPy package.(Jones et al., 2001; Walt et al., 2011) This permits interoperation of Java and Python, enabling seamless links between the two environments. The Jython (Python implemented in Java) interpreter(Hugunin et al.; Pedroni & Rappin, 2002) comes preconfigured in DAWN, with basic NumPy functionality made available through the `Dataset` class and its associated libraries, via the ScisoftPy Python module.(Chang, 2010) GDA incorporates the same Jython environment; thus data processing scripts to be used with GDA may be developed and tested offline within DAWN prior to the beamtime and without requiring access to the beamline software.
